# Metamorphosis of nursing profession: an Indian perspective

**DOI:** 10.7189/jogh.09.020314

**Published:** 2019-12

**Authors:** Shine Stephen, VR Vijay

**Affiliations:** College of Nursing, All India Institute of Medical Sciences (AIIMS), Bhubaneswar, India

After World War II, the United States suffered an intense shortage of physicians. There emerged an intense imbalance between supply and demand of health care facilities. Medical facilities were inadequate to meet the demands of people, especially rural population. The available health care facilities were inaccessible to many of the poorest Americans in terms of increased cost. People resided in the interior areas of cities and rural countryside were badly in need of medical assistance. In order to tackle this physician scarcity, they trained qualified and skilled nurses to practice as nurse practitioners.

In high-income countries like UK, Ireland, USA, Netherlands, Australia, Canada and Finland, and even in some middle- and low-income countries such as Thailand and Nigeria, nurse practitioners are being used independently [1]. From the data across the globe, it is evident that nurse practitioners are the first point of contact for patients in health care system. The patient satisfaction with nurse practitioners is also seen more or as equal to that of a physician. With successful implementation of the nurse practitioners the expense on health care has also been reduced in many countries. A lot of studies have found that there is no difference between nurse practitioners and physician in clinical outcomes [2,3].

A nurse practitioner (NP) is a registered nurse with advanced academic and clinical experience, which enables them to diagnose and manage most common and many chronic illnesses, either independently or as part of a health care team [4].

Latest reports shows that 68.85% of Indian population resides in rural areas [5]. About 59.2 percentage of health workers resides in urban area where only 31.15 percentage of the whole population resides [6]. Incidence of all diseases in rural population is double when compared to those from urban population. Usually rural people do not have access to proper health care, since it is manly intended to serve the well off [7]. The Indian urban middle class have access to health services that is in par with international standards, while at least bottom level health care facilities are intangible to 135 million people in the rural and tribal areas. Even though some services are provided; it is of inferior quality by untrained personnel [7]. Presently India is experiencing acute shortage of physicians especially in the rural areas. It is reckoned that there is one physician per 1300 population. This is far beyond the WHO recommendation of one physician per 1000 population. This is much below when compared to other countries also.

Recent report shows about 27% of doctors in primary health centres (PHC)and more than half of specialists post in community health centres (CHC) remains vacant.

As per the latest reports, almost one third of sanctioned posts of doctors in PHC and nearly half of the sanctioned posts of specialists in CHC remain vacant. This throws light to the utmost need of Nurse Practitioners in country like India (**Table 1**).

**Table 1:** Vacant posts of doctors and specialists in India*

Posts of doctors and specialist in health centres in India		
	**Sanctioned**	**Vacant**
Doctors in PHC	34 750	9389
Specialists in CHC	11 661	7881

PHC – primary health centres, CHC – community health centres

*Source: Rural Health Statistics, Govt. of India; Ministry of Health and family welfare [8].

Due to the above-mentioned reasons, the need of Nurse Practitioner in India is inevitable. Moreover, the National Health Policy put forward by Ministry of Health and Family Welfare, Government of India 2017 signifies the importance of Nurse practitioners to meet vivid health care needs in different areas [9].

## ROLE OF NURSE PRACTITIONERS

According to a National Survey of Nurse Practitioners conducted by the Health Resources and Services Administration (HRSA) Bureau of Health Professionals National Centre for Health Workforce Analysis, New York, the role of nurse practitioners are;

- Counsel and educate patients and families,
- Conduct physical examinations and obtain medical histories,
- Prescribe drugs for acute and chronic illnesses,
- Order, perform and interpret lab tests, x-rays, ECGs and other diagnostic studies,
- Diagnose, treat and manage acute illnesses,
- Diagnose, treat and manage chronic illnesses,
- Provide preventive care, including screening and immunizations,
- Provide care coordination,
- Make referrals,
- Perform simple procedures.

## SCOPE OF NURSE PRACTITIONERS

Scope lies beyond the level of a registered nurse. They can work autonomously or in association with other health care professionals, which help in promoting health, preventing disease and improving access to health outcomes. Nurse practitioners gather higher-level knowledge and skills in nursing and apply in diagnosing and provide therapeutic care to the patient. They contribute an immense range of assessment and therapeutic interventions, prescribing and interpreting diagnostic and laboratory tests, prescribing medications within their range of knowledge and proficiency. They are competent to make admission and discharge from a health care facility as and when needed. Nurse practitioners can also contribute to research in the profession which helps in uplifting the quality of care.

Nurse practitioners can play an active role starting from the primary level care in the community to hospital care level both secondary and tertiary levels. They can take a public health leadership role in health care sector of India by studying the success stories from developed countries [10]. There should be strong and sound back up from the government to implement nurse practitioner program in India.

## IMPLEMENTATION OF NURSE PRACTITIONER PROGRAM IN INDIA

**Figure 1:**
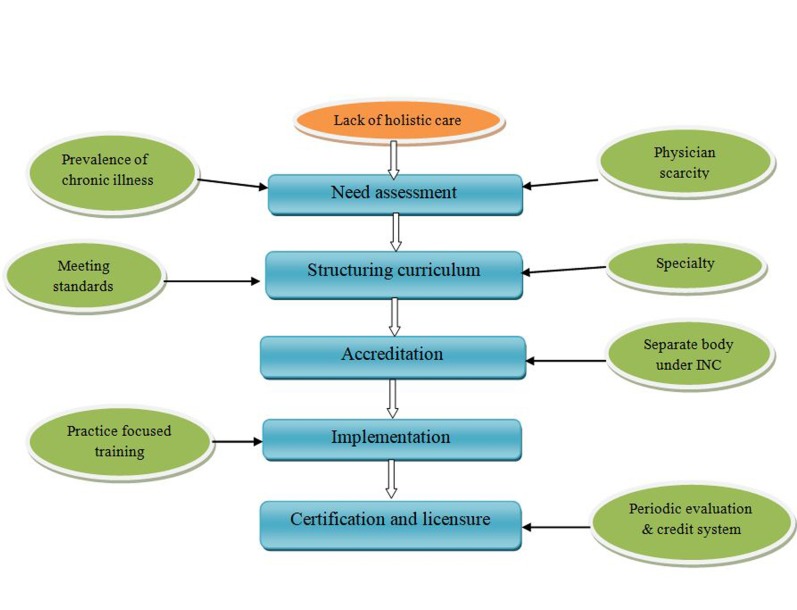
Implementation of NP program in India.

The following things should be considered before implementing nurse practitioner program in India (**Figure 1**):

1. Need assessment: Preliminary need assessment of program is to be done. This must focus on physician scarcity in rural area, prevalence of chronic illnesses, increased life expectancy and lack of holistic care if provided by physicians. Care delivery by nurse practitioner will be holistic.
2. Structuring curriculum: The curriculum should be in par with international standards. Different speciality viz. geriatrics, adult care, maternal care, paediatric care, medical, surgical care should be initiated.
3. Accreditation: A separate body can be formed under INC, which includes specialists in nurse practitioner from abroad, nursing experts, and nursing faculty from national institutes. Accreditation should be given by that separate body.
4. Implementation: More focus should be given for assessing and identifying symptoms, patient assessment, proper history collection, prescribing for specific diagnostic measures and diagnosis. For this frequent practical session must be arranged in presence of experts.
5. Certification and licensure: Periodic evaluation must be done by arranging practical sessions and structured exams. Licensure should be renewed periodically via credit system.
6. Benefits of nurse practitioner: Nurse practitioner gives society and self many benefits. It improves the quality of care delivered as well as enhance the confidence of the care-giver.

## BENEFITS TO SOCIETY

NPs play a critical role in uplifting the health status of people in the society. The health status will be improved in terms of preventive as well as curative aspects.

### Preventive care

Physician often treat patients once a disease occurs, but the nurse practitioner monitors the health status and lifestyle of patients from a preventive aspect. This curtails the initiation and progression of disease. Mostly NPs work under the supervision of medical experts which aids her for expert opinion and appropriate referrals as and when needed.

### Specialization

NPs gets ample opportunity to get expertise in their area of interest such as cardiology, oncology or geriatrics similar to medical professionals. This sort of specialization suits to deliver high quality and comprehensive care to the patients. More over specialization in a particular area throws light towards lots of research activities.

## Benefits to NP’s

### Career growth

NP’s deliver many services more or less similar to the physicians. As they are getting experienced, high quality care can be provided which makes them more professional and acceptable in the society. The job profile will be changed from mere bed-side care provider to highly skilled and proficient decision maker.

### Autonomy

Autonomy of work cannot be enjoyed if nurses work in any other areas. NP’s can do their own practice and enjoy a high degree of autonomy. A high degree of control over the work can be achieved which improves work quality [11].

### Advanced nursing

Practice of NP does rely not just in diagnosing and treating specific disease conditions. They create integrated health care plans that provide holistic care to the patients. Thus, they become familiar with patients and develop a good interpersonal relationship. It allows them to frequently monitor individual health and treatment plans over a long term [12].

### Higher profile role

An NP holds an esteemed position between a registered nurse and a doctor. Albeit most NP’s hold a master’s degree, they have the benefit of bypassing the broad and expensive nature of restorative school training and internships required for doctors. This enables a NP to launch her career in a shorter time range.

## CHALLENGES AND RESOLUTIONS OF NP IN INDIA

With no doubt NP became mandatory in our health care system. Certainly, obstacles are inevitable but the concern is how the policy makers will intervene those. There are certain constrains which should be addressed before jumping to NP. The hurdles could be many but importantly certain questions could have raised: who will be the faculty and what are the required qualifications for them, where will be the educational set up, who will make the curriculum, where the students will undergo training, where they will practice after completing the course, whether the medical fraternity will allow for the same, whether any existing law will support their independent practice or inculcation or amendment is needed?

At present, in India the existing laws are not allocating freedom to practice independently for the nurses. So, amendment of nursing act should be done to make it practical. Solid evidences from USA are available to be adapted. Indian nursing council must amend the existing nursing practicing act that will enable the registered NP to practice independently. Resolution of existing challenges should start from the curriculum development. At present, seemingly interest to point out that in India, none of the faculty are capable to run the NP course because they hadn’t trained to do so. Hence fore, as a solution, the concerned selected faculty from National or esteemed institute should be trained either to send them abroad or to bring eminent NP from western countries to India. A faculty exchange programme will be worth enough in this particular scenario. Well experienced and trained national or international faculty is a must criteria that we need to have with us before starting the course.

**Figure:**
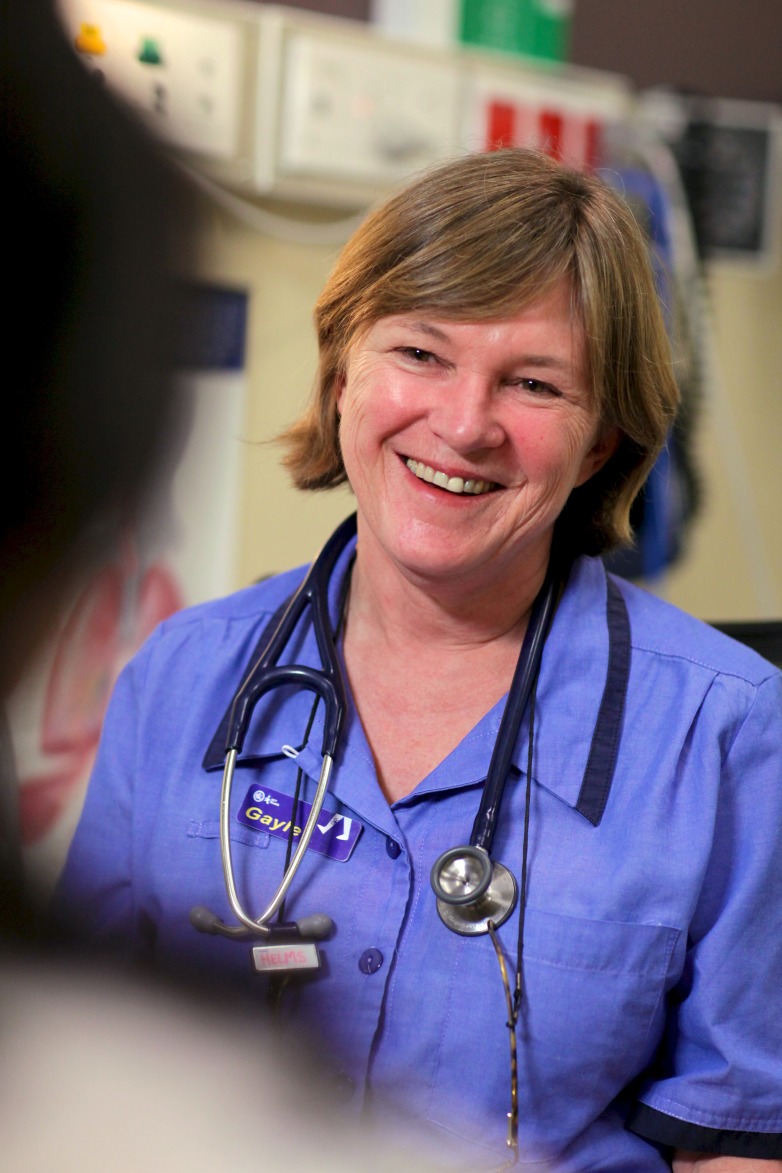
Photo: Nurse practitioner Gayle Comyn consults with a patient. The Canberra Walk-in Centre is Australia's first nurse-led walk-in cliniDIAC images [CC BY 2.0 (https://creativecommons.org/licenses/by/2.0)].

Political willingness and support must be gained before leaping to NP. To surpass medicos’ arguments of projecting the incapability of nursing fraternity to perform independently, we need to assimilate the evidences which could be an accelerator in our efforts towards the accomplishment of ultimate goal (NP). So a well-planned leap is mandatory and too the need of the hour. To train the NP students, getting participation of hospital administration and physician are also important as they feel a threat from NP in their future endeavour. Govt of India has to forward clear directions to all National institutes to initiate and facilitate the smooth running of NP training programs in their setup. Ministry of health and family welfare should create well designed faculty post before starting the course. It should be formulated as a residency programme with proper blend of theoretical and practical knowledge and skill and the NP students/ trainees should get sufficient residency stipend during their course.

Now the question is where they will practice once they complete the course successfully, because as of now in India, there is no special post for NP to practice. Govt of India to create posts to accommodate the successfully trained NPs in the health care sector both in service and education arena. While creating such posts, there should be clear job responsibilities, scope of their work, priori registration, clear and linear recruitment criteria, payment and other allowances, clear promotional strategies etc, should be established for their transparent and goal oriented practice as par with western countries. Also anticipate the delay in mobilising and convincing the public to seek service from the NP as it will take time to establish a trust in NPs and to accept them as their primary health care practitioner. There is no doubt in the success of NP in all aspects as it really intended to achieve, if it establishes with real goals and stringent criteria and visionary guidelines.

## NURSE PRACTITIONER PROGRAM INITIATED IN INDIA

After the recommendation of National Health Policy 2015; Indian Nursing Council (INC) has put forwarded an agenda to initiate Nurse Practitioner program in Critical Care. Based on the curriculum advised by INC, some institutions are running post graduate residency program on nurse practitioner in critical care. Some specialities like acute care, gerontology, neonatology, paediatrics, psychiatry are still lacking.

## NURSE PRACTITIONERS IN FUTURE

The prospects of nurse practitioner include independent nurse practitioner run clinics, governmental norms that accept nurse practitioners as primary care providers, deploy nurse practitioners to outreach clinics and industries to provide emergency care, to treat a variety of episodic diseases such as urinary tract infections and conjunctivitis. The drawback in these clinics are, even though they provide exposure for nurse practitioners to the general public the patients tend to be referred back to their primary care provider if the nurse practitioner identifies a complicated problem. In order to tackle this problem, the nurse practitioner should be skilled enough to manage complications also. Curriculum should be implemented accordingly.

A four year doctor-of-nursing-practice program can be started which will incorporate clinical studies and to initiate independence training.

Nurse practitioner is a vital part of transdisciplinary health care team. An aggregation of medical skills and nursing care provide a unique role which fill gap in numerous health care settings, which further improves patient outcomes [13].

## CONCLUSIONS

It is very evident that NPs are need of the hour. Developed western countries had framed and implemented NP programme that is continuing its triumph despite of the resistance of physician groups. With such good patient-physician ratio, if such countries can run NP programme then why not in developing countries like India? Where millions are still lacking primary health care. The panoramic impacts of NPs are cost-effectiveness, accessibility, holistic and patient centred approach, further it cater the health care needs of the subservient rural population.
